# Traumatic reticuloperitonitis combined with embolic pneumonia and hepatitis as unusual symptoms of foreign body syndrome in a Holstein bull

**DOI:** 10.1002/vms3.341

**Published:** 2020-08-18

**Authors:** Verônica L. de Souza, Arthur M. S. V. Pinto, Cintia R. R. Queiroz, Márcio B. de Castro, José R. J. Borges, Benito Soto‐Blanco, Antônio C. L. Câmara

**Affiliations:** ^1^ Large Animal Veterinary Teaching Hospital College of Agronomy and Veterinary Medicine Universidade de Brasília Brasília DF Brazil; ^2^ Veterinary Pathology Laboratory College of Agronomy and Veterinary Medicine Universidade de Brasília Brasília DF Brazil; ^3^ Department of Veterinary Clinics and Surgery Veterinary College Universidade Federal de Minas Gerais Belo Horizonte MG Brazil

**Keywords:** embolic pneumonia, foreign body syndrome, hardware disease, traumatic reticuloperitonitis

## Abstract

Traumatic reticuloperitonitis combined with embolic pneumonia and hepatitis is unusual signs of foreign body syndrome in cattle. A 4‐year‐old Holstein bull presented decreased appetite, dry cough, progressive weight loss, sternal recumbence and reluctance to stand and move. Laboratory tests revealed leucocytosis (18.4 × 10^3^/μl) accompanied by neutrophilia (10.48 × 10^3^/μl), and monocytosis (1.28 × 10^3^/μl), hyperglobulinaemia (6.3 g/dl), hypoalbuminaemia (1.5 g/dl), hyperfibrinogenaemia (10 g/L) and severe increase in gamma‐glutamyl transferase activity (1,216 U/L). Reticular ultrasonographical examination showed a large amount of hyperechoic and hypoechoic content between the reticular serosa and the hepatic visceral surface. The main gross findings included fibrin deposition and adhesions between the reticulum, liver and diaphragm surfaces; a 4.0 mm in diameter transmural reticular perforation; a 12.0‐cm diameter and scarce small randomly abscesses in the liver's parenchyma. The lungs presented multifocal areas of suppurative embolic foci (pulmonary abscesses), interstitial emphysema and multifocal fibrin deposition on the pleural surface. Ancillary diagnostic tests, such as ultrasonography and laboratory test, associated with clinical evaluation, may increase the accuracy of the correct diagnosis and avoid wasting time and money on untreatable cases.

## Graphical abstract

Foreign body syndrome may cause a wide variety of clinical signs and represents a challenge for practitioners, especially when multiple organs are involved, increasing the difficulty in early prediction of potential sequel, based only on clinical presentation. This manuscript describes for the first time the occurrence of traumatic reticulohepatitis and parareticular abscessation causing embolic pneumonia and pleuritis.

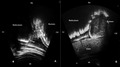

## 1 INTRODUCTION

Cattle and buffaloes are prone to ingest foreign objects, mainly due to erroneous management practices and lack of discriminatory dietary habits (Roth & King, 1991). When the swallowed foreign bodies are metallic objects, most commonly wires and nails, they may reach the reticulum and become trapped within the reticular honeycomb‐like mucosa. This condition is known variously worldwide as foreign body syndrome (FBS), hardware disease, sharp foreign body syndrome or traumatic reticuloperitonitis (Abu‐Seida & Al‐Abbadi, 2016). Vigorous reticular contractions facilitate penetration of the reticular mucosa, causing various syndromes depending upon the length and direction of these objects (Khalphallah et al., 2017). FBS may result in several complications such as reticulitis, localized or diffuse reticuloperitonitis and pericarditis (Braun, Lejeune, Schweizer, Puorger, & Ehrensperger, 2007; Braun, Warislohner, Torgerson, Nuss, & Gerspach, 2018; Dirksen, 2005).

Unfortunately, FBS remains one of the most common digestive disorders of cattle and buffaloes, especially in developing countries, mostly due to poor management practices (Khalphallah et al., 2017). Although there are numerous reports on FBS affecting these species (Abu‐Seida & Al‐Abbadi, 2016; Braun et al., 2007, 2018; Gerspach, Wirz, Schweizer‐Knubben, & Braun, 2011; Roth & King, 1991; Watts & Tulley, 2013), few published reports have discussed how this syndrome affects other organs such as the lungs, spleen and liver (Dirksen, 2005). A review of the literature found articles discussing pathological features in sporadic cases of metallic foreign bodies embedded in the liver (Abu‐Seida & Al‐Abbadi, 2016; Ismail & Abdullah, 2014; Roth & King, 1991), but these reports lack clinical, laboratory and ultrasonography findings. Furthermore, concomitant traumatic reticuloperitonitis and bronchopneumonia (Braun, 2008), or hepatic abscesses (Braun, Pusterla, & Wild, 1995) have been reported in cattle, but no information is available regarding direct penetration of the foreign bodies on the liver. Therefore, this paper aims to describe clinical, laboratory, ultrasonographical and pathological findings of traumatic reticuloperitonitis combined with secondary embolic pneumonia and hepatitis in a Holstein bull. Additionally, this report also highlights ancillary diagnostic tests, such as ultrasonography and laboratory tests, associated with clinical evaluation to avoid wasting time and money on untreatable cases.

## 2 CASE HISTORY

A 4‐year‐old Holstein bull was referred for veterinary evaluation at the Large Animal Veterinary Teaching Hospital, University of Brasília, Distrito Federal, Midwestern Brazil. The owner reported that the bull had decreased appetite, dry cough and progressive weight loss for one week. Three days before it was presented at the clinic, the bull had been founded grunting in sternal recumbence with reluctance to stand and move. The bull did not receive any medications on the farm.

Clinical examination was performed (Dirksen, Gründer, & Stöber, 1993), and revealed apathy, a moderate body condition score (graded as 3/5), dehydration with enophthalmos, normal heart rate (72 beats/min), tachypnea (64 breaths/min) with mixed dyspnoea and crackles, sporadic dry cough, elbow abduction and ruminal and intestinal hypomotility. Rectal body temperature was 38°C. Reticular foreign bodies’ clinical tests were conducted, such as pinching of the withers, upward pressure on the xiphoid area and reticular percussion. The bull elicited a positive response only in the withers pinch test. Ororuminal probe passing was unremarkable. The ruminal fluid analysis revealed increased pH (8.0), and rumen fluid was considered inactive (>6 min) based on the results of the methylene blue reduction test (Braun et al., 2018; Dirksen et al., 1993).

Haematological analysis revealed leucocytosis (18.4 × 10^3^/μl; reference range: 4–12 × 10^3^ leucocytes/μL) accompanied by neutrophilia (10.48 × 10^3^/μl; reference range: 0,6–4 × 10^3^ neutrophils/μl), and monocytosis (1.28 × 10^3^/μl; reference range: 0.025–0.84 × 10^3^ monocytes/μl) (Wood & Quiroz‐Rocha, 2010). Serum biochemistry profile showed hyperglobulinaemia (6.3 g/dl; reference range: 3.0–3.48 g/dl), hypoalbuminaemia (1.5 g/dl; reference range: 3.03–3.55 g/dl), hyperfibrinogenaemia (10 g/L; reference range: 2–7 g/L) and severe increase in gamma‐glutamyl transferase activity (GGT) (1,216 U/L; reference range: 6.1–17.4 U/L). Urea level, creatinine level and aspartate aminotransferase activity were within reference limits (Kaneko, Harvey, & Bruss, 2008).

**FIGURE 1:**
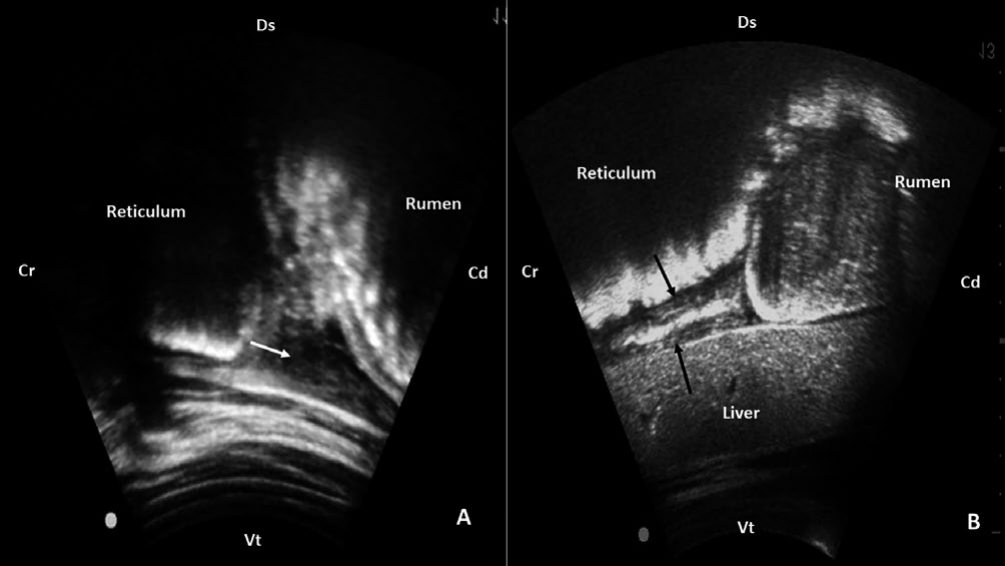
Ultrasonography of a Holstein bull showing traumatic reticulohepatitis. (a) Note that the reticulum serosa is slightly irregular with attachment of echogenic deposits; there is a moderate amount of free anechoic fluid in the abdominal cavity, and a large number of hypoechoic filaments adhering to the surfaces of adjacent organs (white arrow). (b) Presence of hyperechoic and hypoechoic content between the reticular serosa and the hepatic visceral surface (black arrows). Cr: cranial; Cd: caudal, Ds: dorsal, Vt: ventral

Ultrasonographical examination was performed on the reticulum and surrounding areas using a 3.5‐MHz convex transducer from the 6th to 8th intercostal spaces. On the paramedian region to the left of the xiphoid process, the caudoventral reticular wall was found to has slightly irregular serosa and attachment of echogenic deposits. Additionally, there was a moderate amount of free anechoic fluid in the abdominal cavity with a large amount of hypoechoic filaments adhering to the surfaces of adjacent organs (Figure 1a, white arrow). During biphasic contractions, the reticulum contracts by only 1–3 cm, suggesting adhesions (Braun, 2003). On the right paramedian region, hyperechoic and hypoechoic content between the reticular serosa and the hepatic visceral surface was noted, suggesting fibrinous adhesions between these organs (Figure 1b, black arrows). Thoracic and reticular radiographs were taken, but the low potency of our equipment precluded useful radiographs imaging.

The bull evolved to more pronounced elbow abduction, frequent vocalization, head and neck extension with open‐mouth breathing within 4‐hr. Based on clinical, laboratory and specially ultrasonographical findings, supporting the presumptive diagnosis of traumatic reticulohepatitis, euthanasia was elected (0.1 mg.kg^‐1^ of xylazine followed by sodium thiopental overdose intravenously). A post‐mortem examination was then conducted.

**FIGURE 2:**
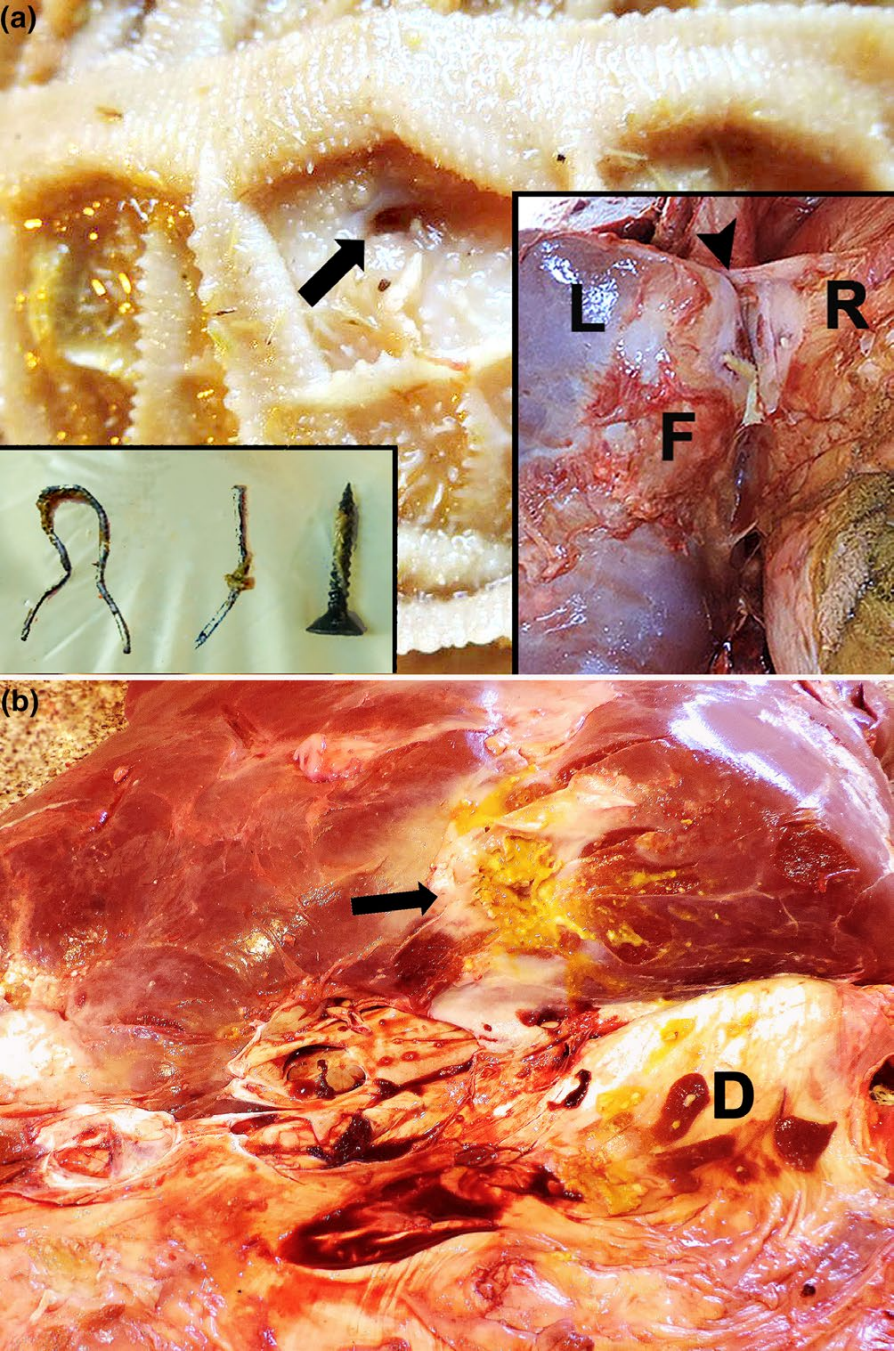
Gross findings, Holstein bull. (A) Circular transmural perforation with dark edges in the reticulum (arrow). Close views: Left—metallic foreign bodies found within reticulum; Right—fibrosis and yellow suppurative material at the abscess site (head arrow) in the liver (L), fibrin deposition (F) and adhesions with the reticulum (R). (B) Liver abscess filled with yellow purulent content surrounded by fibrosis (black arrow) at the diaphragmatic surface (D)

**FIGURE 3:**
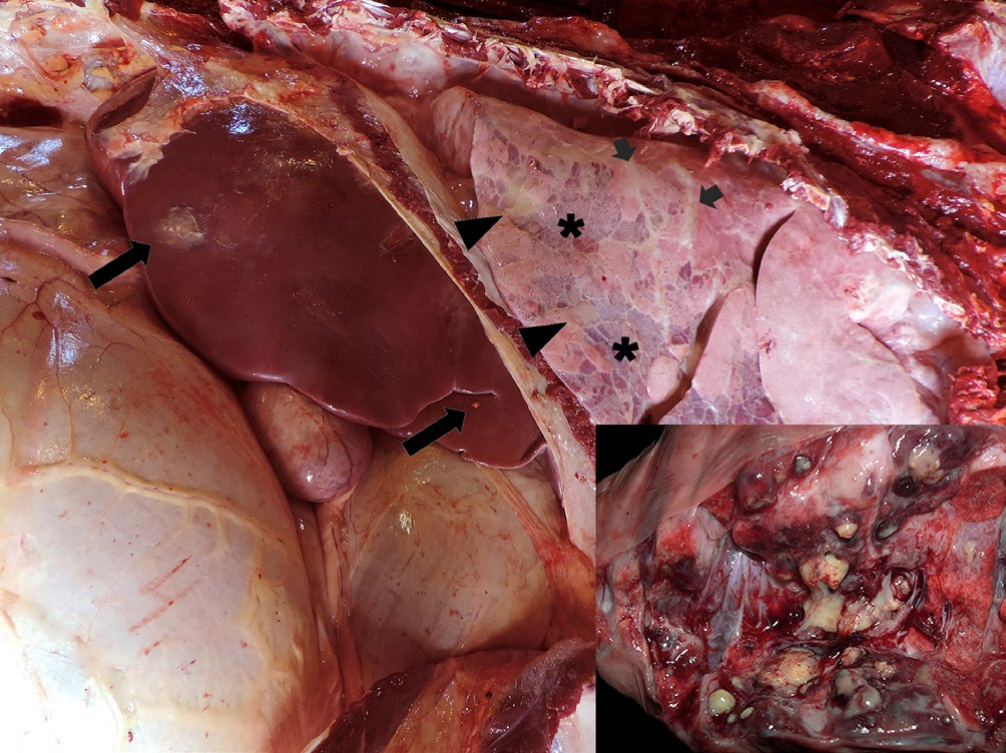
Abdominal and thoracic organs, Holstein bull. Embolic pneumonia and pleuritis with fibrin deposition on the pleural surface (arrow heads), suppurative pneumonic foci (asterisks), interstitial emphysema (short arrows) and small randomly distributed liver abscesses (long arrows). Close view, lung, cut surface: pulmonary embolic abscesses surrounded by hyperaemic areas

**FIGURE 4:**
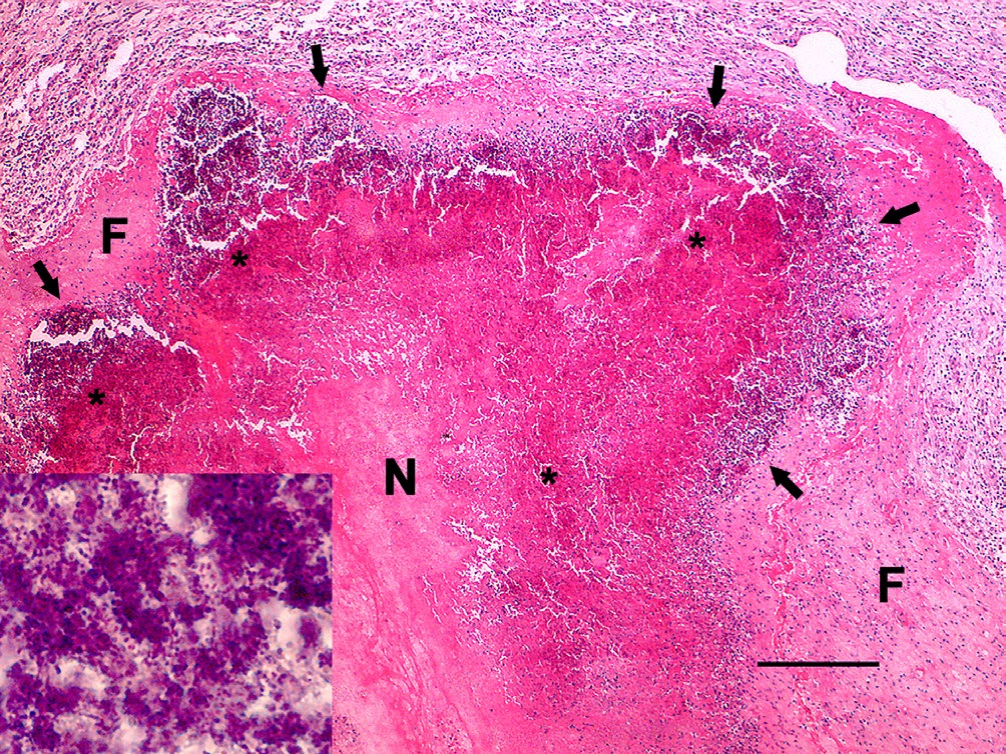
Lung, Holstein bull. Embolic pulmonary abscessation secondary to traumatic reticulohepatitis with a necrotic centre (N) within bacterial myriads (asterisks), lined by neutrophilic inflammatory infiltrate (arrows) surrounded by a fibrous capsule (F) (H&E, bar = 250 µm). Close view: Bacterial myriads composed by a mixed of bacilli and cocci (Gram stain)

Fibrin deposition and adhesions between the reticulum, liver and diaphragm surfaces were the main gross findings in the abdominal cavity. A circular 4.0 mm in diameter transmural perforation with darkened edges was detected at the cranial region of the reticulum, in contact with the diaphragm surface and liver (Figure 2a). Free perforating metallic bodies (wire, screws and nails), ranging from 3–8 cm in length, were found mixed with food content (Figure 2a). The liver presented an abscess (12.0‐cm diameter) in the left lobe filled with yellow purulent material adhered with the diaphragm and reticulum in continuity to the perforation site and surrounded by marked fibrosis (Figure 2b). Additionally, scarce small randomly abscesses, distributed in the liver's parenchyma, were also detected (Figure 3). The lungs presented multifocal areas of inflammatory parenchymal consolidation associated with suppurative embolic foci (pulmonary abscesses), interstitial emphysema and multifocal fibrin deposition on the pleural surface (Figure 3). Microscopically, necrosuppurative foci in the liver and diaphragm close to the perforation site and embolic multiple abscesses in the lungs and liver were detected. Abscessations presented necrotic centres within bacterial myriads (Gram‐positive and ‐negative bacillus and cocci aggregates) lined by a neutrophilic inflammatory infiltrate and surrounded by a fibrous capsule (Figure 4). In the lungs’ surface, we also detected aggregates of fibrin. No other relevant gross or microscopic changes were observed in other organs (brain, heart, kidneys, spleen or other abdominal and thoracic organs and tissues).

## 3 DISCUSSION

Foreign body syndrome (FBS) is observed most commonly in developing countries due to poor management practices. FBS incidence in animal hospitals of Asian and African countries may vary from 22.9% to 59.14% in cattle and buffaloes (Abu‐Seida & Al‐Abbadi, 2016; Khalphallah et al., 2017). In Brazil, only two retrospective studies on digestive diseases have been found in the literature; these presents prevalence of 3.16% (19/600) and 17.2% (40/233) in cattle from Southern (Mello et al., 2017) and Northeastern (Marques et al., 2018) regions, respectively. The real economic impact of this syndrome on the Brazilian cattle industry may be being overlooked.

Clinical signs of traumatic reticuloperitonitis depend upon the site of reticular perforation and the lesions on the surrounding areas and organs involved (Braun et al., 2003, 2018); most common signs include apathy, anorexia, decreased milk production, fever, reluctance to move and stance with an arched back and abducted elbows (Abdelaal, Floeck, El Maghawry, & Baumgartner, 2009; Abu‐Seida & Al‐Abbadi, 2016; Braun et al., 2018; Dirksen, 2005; Roth & King, 1991). In cattle with traumatic reticuloperitonitis, the clinical course may vary from sudden death to months (Roth & King, 1991), and in contrast, cattle with liver abscesses can be asymptomatic, or reduced feed intake and efficiency may be evident (Braun et al., 1995; Doré, Fecteau, Hélie, & Francoz, 2007). In this report, the bull's severe systemic disturbances resulted from digestive, respiratory and hepatic involvement.

Although radiography is considered to be an efficient technique for identifying the location and position of metal foreign bodies in cattle or buffaloes (Abu‐Seida & Al‐Abbadi, 2016; Khalphallah et al., 2017), ultrasonography is considered to be the method of choice for detecting fibrinous deposits and abscesses that usually are not detected by radiographical examination (Abdelaal et al., 2009; Braun, 2003). In this report, ultrasonographical examination showed shortened biphasic reticular contractions and inflammatory material deposits between the reticular, ruminal and hepatic visceral surface. Herein, we are additionally reporting the relevance of ultrasonographic evaluation on reticulohepatitis sequelae, such as abdominal effusion and adhesions between the reticulum and liver. Alternatively, an ultrasonographical examination can provide information about the extent of the lesions and select the best suitable site for abdominocentesis (Abu‐Seida & Al‐Abbadi, 2016; Braun, 2003; Ellah, El‐Hawari, Misk, Youssef, & Semieka, 2018; Khalphallah et al., 2017). Although thoracic ultrasonography has not been performed, it would add essential data reiterating the bull's euthanasia choice. Pulmonary abscesses are usually imaged at 3rd and 4th intercostal spaces as circumscribed masses with echogenic capsule and hypoechoic contents, but only pulmonary abscesses near the pleura can be imaged by ultrasound (Abu‐Seida & Al‐Abbadi, 2016).

Severe neutrophilic leucocytosis, hyperfibrinogenaemia and hyperglobulinaemia are consistent with bacterial infection. No immature leucocyte was found in smears, thus increased neutrophils counts might be attributed to movement of these cells from marginal pool to the circulating pool (Wood & Quiroz‐Rocha, 2010). Hypoalbuminaemia occurred because albumin is a negative acute‐phase protein, and it might be worsened by malnutrition and improper digestion due to pain and adhesions (Braun et al., 2018; Ellah et al., 2018). The highly elevated GGT activity (1,216 U/L) in this case was a remarkable biochemical finding; previously reported values vary from 16–256 U/L (Doré et al., 2007) and 7–73 U/L (Braun et al., 2007) to ≤154 UI/L (Braun et al., 2018) in cattle affected by liver abscesses, traumatic reticuloperitonitis or pericarditis, respectively. Serum GGT activity is a specific indicator of liver disorders because this enzyme is released by biliary ducts cells and, in lesser amounts, by hepatocytes (Kaneko et al., 2008); however, increases in GGT activity in cases of traumatic reticuloperitonitis (Braun et al., 2018) and pericarditis in cattle (Braun et al., 2007) or buffaloes (Ellah et al., 2018) are associated with liver congestion and not primary liver disease (Braun et al., 2007). In our case, multiple liver abscessation may had compressed, and partially obstructed adjacent bile ducts and canaliculi, resulting in markedly increased GGT activities.

Liver abscess in extension with the traumatic reticuloperitonitis observed in the Holstein bull, possibly led to bacterial spread and embolic septic dissemination to the liver and lungs. Traumatic reticuloperitonitis with abscessation of the reticulum wall are associated with bacteraemia and infection dissemination to the liver, lungs and other organs (Watts & Tulley, 2013) as a result of embolus formation into hepatic veins, leading to an acute fatal outcome (Cullen & Stalker, 2016). Despite we did not find a metallic body embedded in the liver or in the perforation, nails, screws and wires were detected in the reticular lumen. Possibly, reticular contractions pushed the metallic foreign bodies through the mucosa reaching the liver, allowing abscessation, adhesions and fibrosis. Reticular foreign bodies trapped in the reticular mucosa could also penetrate a mucosal fold or pierce the reticular wall (Dirksen, 2005; Farrow, 1999).

Random abscesses in the liver and pulmonary parenchyma supports the hypothesis of bacterial spreading from the primary septic focus. Direct or embolic infection dissemination is a common sequel in cattle with traumatic reticuloperitonitis (Cullen & Stalker, 2016; Roth & King, 1991) and may lead to other complications such as fibrinous inflammation and fibrous adhesions to adjacent viscera (Roth & King, 1991), septic arthritis and endocarditis (Watts & Tulley, 2013). In our case, septic emboli from the liver abscesses reached the lungs, resulting in multifocal embolic pneumonia and pleuritis (Braun, 2008; Gerspach et al., 2011).

Definitive diagnosis of traumatic reticulohepatitis and parareticular abscessation causing embolic pneumonia and pleuritis was established on the basis of clinical, laboratory, ultrasonographical and pathological findings. Foreign body syndrome may cause a wide variety of clinical signs and represents a challenge for practitioners, especially when multiple organs are involved, increasing the difficulty in early prediction of potential sequel, based only on clinical presentation. Ancillary diagnostic tests, such as ultrasonography and laboratory test, associated with clinical evaluation may increase the accuracy for correct diagnosis, and avoid wasting time and money on untreatable cases.

## CONFLICTS OF INTEREST

The authors have no conflict of interest.

## AUTHOR CONTRIBUTION

**Verônica L.ça de Souza:** Investigation; Writing‐review & editing. **Arthur M. S. V. Pinto:** Investigation; Writing‐review & editing. **Cintia Regina Rêgo Queiroz:** Investigation; Writing‐review & editing. **Márcio Botelho de Castro:** Data curation; Investigation; Writing‐review & editing. **José Renato Junqueira Borges:** Investigation; Writing‐review & editing. **Benito Soto‐Blanco:** Investigation; Writing‐review & editing. **Antônio Carlos Lopes Câmara:** Conceptualization; Data curation; Investigation; Writing‐original draft; Writing‐review & editing.

## ETHICAL STATEMENT

The authors confirm that the ethical policies of the journal, as noted on the journal's author guidelines page, have been adhered to. No ethical approval was required as this is an investigation of an animal at post‐mortem examination.

### PEER REVIEW

The peer review history for this article is available at https://publons.com/publon/10.1002/vms3.341.
